# Predictive value of progression-related gene classifier in primary non-muscle invasive bladder cancer

**DOI:** 10.1186/1476-4598-9-3

**Published:** 2010-01-08

**Authors:** Wun-Jae Kim, Eun-Jung Kim, Seon-Kyu Kim, Yong-June Kim, Yun-Sok Ha, Pildu Jeong, Min-Ju Kim, Seok-Joong Yun, Keon Myung Lee, Sung-Kwon Moon, Sang-Cheol Lee, Eun-Jong Cha, Suk-Chul Bae

**Affiliations:** 1 Department of Urology, College of Medicine, Chungbuk National University, Cheongju, Chungbuk, South Korea; 2 BK21 Chungbuk Biomedical Science Center, School of Medicine, Chungbuk National University, Cheongju, Chungbuk, South Korea; 3 School of Electrical and Computer Engineering, Chungbuk National University, Cheongju, Chungbuk, South Korea; 4 Department of Food and Biotechnology, Chungju National University, Chungju, Chungbuk, South Korea; 5 Department of Biomedical Engineering, College of Medicine, Chungbuk National University, Cheongju, South Korea; 6 Department of Biochemistry, College of Medicine, Chungbuk National University, Cheongju, South Korea

## Abstract

**Background:**

While several molecular markers of bladder cancer prognosis have been identified, the limited value of current prognostic markers has created the need for new molecular indicators of bladder cancer outcomes. The aim of this study was to identify genetic signatures associated with disease prognosis in bladder cancer.

**Results:**

We used 272 primary bladder cancer specimens for microarray analysis and real-time reverse transcriptase polymerase chain reaction (RT-PCR) analysis. Microarray gene expression analysis of randomly selected 165 primary bladder cancer specimens as an original cohort was carried out. Risk scores were applied to stratify prognosis-related gene classifiers. Prognosis-related gene classifiers were individually analyzed with tumor invasiveness (non-muscle invasive bladder cancer [NMIBC] and muscle invasive bladder cancer [MIBC]) and prognosis. We validated selected gene classifiers using RT-PCR in the original (165) and independent (107) cohorts. Ninety-seven genes related to disease progression among NMIBC patients were identified by microarray data analysis. Eight genes, a progression-related gene classifier in NMIBC, were selected for RT-PCR. The progression-related gene classifier in patients with NMIBC was closely correlated with progression in both original and independent cohorts. Furthermore, no patient with NMIBC in the good-prognosis signature group experienced cancer progression.

**Conclusions:**

We identified progression-related gene classifier that has strong predictive value for determining disease outcome in NMIBC. This gene classifier could assist in selecting NMIBC patients who might benefit from more aggressive therapeutic intervention or surveillance.

## Background

Bladder cancer is a genetic disorder driven by the progressive accumulation of multiple genetic and epigenetic changes. At the molecular level, these genetic changes result in uncontrolled cell proliferation, decreased cell death, invasion, and metastasis. The specific alterations in gene expression that occur as a result of cross-talk between various cellular pathways determine the biologic behavior of the tumor, including growth, recurrence, progression and metastasis, and may influence patient's survival. While several molecular markers for the development, recurrence and progression of bladder cancer, such as p53 and Rb, have been studied [1-3], the limited value of these established prognostic markers created the need for new molecular indicators of bladder cancer outcomes.

New high-throughput microarray technology makes it possible to gain comprehensive insight into the molecular basis of human diseases [4,5]. With this technology, the RNA expression levels of hundreds or even thousands of genes in a tumor can be surveyed simultaneously. The use of high throughput technologies to assess gene expression patterns in tissues, exfoliated cells in urine, or molecules in serum and in circulating cells for many malignancies, including bladder cancer, has been reported [6,7]. These studies open a door to the possibility of rapidly assessing gene expression patterns in individual tumors to determine tumor classification [8], or to predict clinical outcomes [9,10] and response to chemotherapy [11,12]. In fact, gene expression profiling is currently being tested in clinical trials to define populations of patients with breast cancer who should receive chemotherapy [10,12]. Such trials were launched in Dutch academic centers and in the United States [13].

Many different genetic or epigenetic changes that lead to aberrant gene expression have been identified in bladder cancer [6,7]. Thus, gene expression profiling in bladder cancer represents a potentially useful way to discriminate between good and poor prognosis. Microarray gene expression analysis could be used to facilitate the identification of molecular prognostic markers that correlate with bladder cancer outcomes. In the current study, we identified genetic signatures that are associated with disease progression in patients with non-muscle invasive bladder cancer (NMIBC).

## Methods

### Patients and Tissue Samples

**Figure 1:**
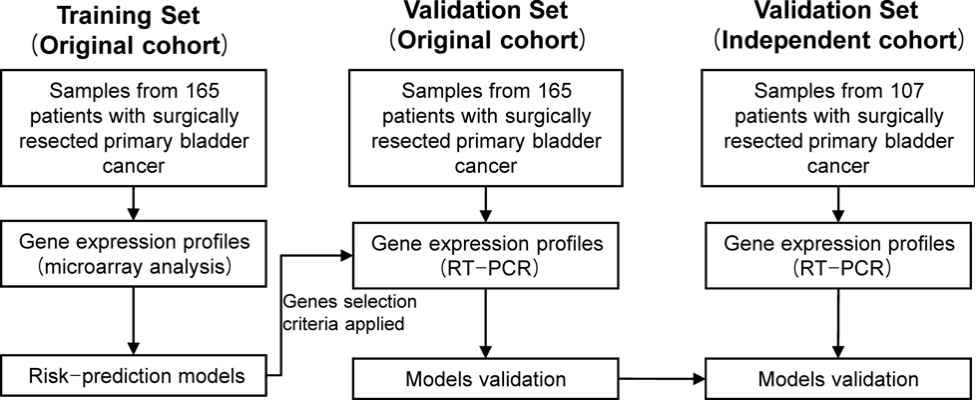
**Study design and validation strategies**.

Table 1 shows the baseline characteristics of the case subjects. We used random computer-generated numbers to assign specimens from 272 consecutive, histologically-verified transitional cell carcinomas in primary bladder cancer patients. To reduce confounding factors for affecting the analyses, any patients diagnosed with concomitant carcinoma *in situ *(CIS) lesion or only CIS lesion were excluded. For the original cohort, we studied the frozen specimens of bladder cancer tissue from 165 randomly selected patients who had undergone surgical resection of a transitional cell carcinoma at the Chungbuk National University Hospital. The mean follow-up period for the original cohort was 48 months (median 37 months; range, 1-137 months). To independently validate our risk-prediction model, 107 randomly selected primary bladder cancer patients who had similar clinico-pathological characteristics and had undergone surgical resection of a transitional cell carcinoma at the same hospital were used as an independent cohort. The mean follow-up period for the independent cohort was 43 months (median, 26 months; range, 1-194 months). The study design and validation strategy are shown in Fig. 1.

**Table 1:** Baseline Characteristics of Primary Bladder Cancer Patients

Variables	Original Cohort (n = 165)	Independent Cohort (n = 107)	*P*
Age - yr (mean)	65.2 ± 12.0	64.1 ± 13.3	0.51*
Gender - no. of patients (%)			0.93^+^
Male	135 (81.8)	88 (82.2)	
Female	30 (18.2)	19 (17.8)	
Grade - no. of patients (%)			0.76^+^
Low	105 (63.6)	70 (65.4)	
High	60 (36.4)	37 (34.6)	
Stage - no. of patients (%)			0.14^+^
NMIBC	103 (62.4)	76 (71.0)	
Ta	23 (22.3)	21 (27.6)	
T1	80 (77.7)	55 (72.4)	
MIBC	62 (37.6)	31 (29.0)	
T2N0M0	26 (41.9)	8 (25.8)	
T3 N0M0	13 (21.0)	8 (25.8)	
T4/Any T N+/M+	23 (37.1)	15 (48.4)	
Recurrence - no. of patients with NMIBC (%)			0.94^+^
No	67 (65.0)	49 (64.5)	
Yes	36 (35.0)	27 (35.5)	
Progression - no. of patients (%)			
NMIBC			0.97^+^
No	92 (89.3)	68 (89.5)	
Yes	11 (10.7)	8 (10.5)	
MIBC			1.00^+^
No	42 (67.7)	21 (67.7)	
Yes	20 (32.3)	10 (32.3)	
Survival - no. of patients with MIBC (%)			
Cancer-specific			0.18^+^
Alive	33 (53.2)	21 (67.7)	
Deceased	29 (46.8)	10 (32.3)	
Overall survival			0.37^+^
Alive	28 (45.2)	11 (35.5)	
Deceased	34 (54.8)	20 (64.5)	
Mean follow-up - months	48.4	42.5	0.25*

Abbreviations: NMIBC, non-muscle invasive bladder cancer; MIBC, muscle invasive bladder cancer.

* *P *value was obtained by Student's t-test.

^+ ^*P *value was obtained by Pearson chi-square test.

All tumors were macro-dissected, typically within 15 minutes of surgical resection. Each bladder cancer specimen was confirmed by pathological analysis of a part of the tissue sample in fresh frozen sections from cystectomy and transurethral resection (TUR) specimens, and then frozen in liquid nitrogen and stored at -80°C until use. The collection and analysis of all samples was approved by the Institutional Review Board of Chungbuk National University, and informed consent was obtained from each subject.

Tumors were staged and graded according to the standard criteria [14,15]. In cases of NMIBC, TUR of the tumor was performed. A second TUR was performed 2-4 week after the initial resection when it was incomplete or when a high-grade or T1 tumor was detected [16]. Patients with intermediate- or high-risk NMIBC received one cycle of intravesical BCG immunotherapy [16,17]. In cases of muscle invasive bladder cancer (MIBC), radical cystectomy and complete pelvic lymph node dissection were performed. Patients with either pT3 or pT4, or node-positive disease, based on the analysis of radical cystectomy specimens, received at least 4 cycles of cisplatin-based chemotherapy. Neither clinically metastatic disease nor non-cystectomy cases were excluded in this study. Each patient has been followed and managed according to the standard recommendation [16-18]. We defined recurrence as the recurrence of primary NMIBC of the same pathologic stage, and defined progression as TNM stage progression after disease relapse in NMIBC and MIBC.

### RNA Extraction

Total RNA was isolated from tissue using the TRIzol reagent (Life Technologies, NY), according to the manufacturer's protocol. The quality and integrity of the RNA was confirmed by agarose gel electrophoresis and ethidium bromide staining, followed by visual examination under ultraviolet light.

### Microarray Gene Expression Profiling

Biotin-labeled cRNA for hybridization was prepared according to Illumina's recommended sample labeling procedure. Briefly, 500 ng of total RNA were used for cDNA synthesis, followed by a coupled amplification/labeling step (in vitro transcription) to synthesize biotin-labeled cRNA using the Illumina^® ^TotalPrep RNA Amplification kit (Ambion Inc., Austin, TX). cRNA concentration was measured using RiboGreen (Quant-iT™ RiboGreen^® ^RNA assay kit; Invitrogen-Molecular Probes, ON, Canada) and a Victor3 spectrophotometer (PerkinElmer, CT). cRNA quality was verified by 1% agarose gel electrophoresis.

Labeled, amplified material (1,500 ng per array) was hybridized to an Illumina Human-6 BeadChip (48K), version 2, according to the manufacturer's instructions (Illumina, Inc., San Diego, CA). Array signals were developed using Amersham fluorolink streptavidin-Cy3 (GE Healthcare Bio-Sciences, Little Chalfont, UK), according to the instructions in the BeadChip manual. Arrays were scanned with an Illumina Bead Array Reader confocal scanner (BeadStation 500GXDW; Illumina, Inc., San Diego, CA), according to the manufacturer's instructions. The full microarray data set is available online http://www.ncbi.nlm.nih.gov/geo/ under the data series accession number GSE13507.

### RT-PCR Analysis

RT-PCR using a Rotor Gene 3000 PCR system (Corbett Research, Mortlake, Australia) was performed in the original and independent cohorts. GAPDH was analyzed in parallel as an internal control. RT-PCR reactions containing primers and SYBR Premix EX Taq (Takara Bio Inc., Otsu, Japan) were carried out in micro-reaction tubes (Corbett Research). Spectral data were captured and analyzed using Rotor-Gene Real-Time Analysis Software 6.0 Build 14 (Corbett Research). Gene expression was normalized to the expression of GAPDH.

### Statistical Analysis

To reduce variation among microarrays, the intensity values for each microarray were rescaled by means of a quantile normalization method [19]. Gene expression values were log2-transformed and median-centered across samples. A hierarchical clustering algorithm, using the uncentered correlation coefficient as the measure of similarity and average linkage clustering, was applied as described in Eisen et al [20].

To select prognosis-related gene classifiers, individual analyses were performed based on tumor invasiveness (NMIBC and MIBC) and prognosis (recurrence, progression, cancer-specific survival and overall survival). Univariate Cox regression analysis for microarray was performed to select genes that correlated significantly with prognosis. In this analysis, we chose a cutoff (P < 0.001) to select candidate genes. Of the candidate prognosis-related gene signatures, genes in the top 10th percentile of gene expression ratios for each classifier were selected for the evaluation of their prognostic properties in the original cohort.

A patient's risk score was calculated as the sum of the levels of expression of each gene multiplied by the corresponding regression coefficients [9,16,21-24]. Patients were classified as having a good-prognosis or poor-prognosis signature, with the 50th percentile (median) of the risk score as the cutoff value [21,23].

To validate the results, RT-PCR analysis of selected genes in the original and independent cohorts was carried out. In the original cohort, genes with a Pearson correlation coefficient of greater than .6 between the microarray and RT-PCR data were selected for validation. Prognostic values were determined by the Kaplan-Meier method, and differences were assessed by log rank statistics. Statistical analysis was performed in R (version 2.6.2), and a P value of < 0.05 was considered statistically significant.

## Results

### Quality assessment of Gene Expression Profile

**Figure 2:**
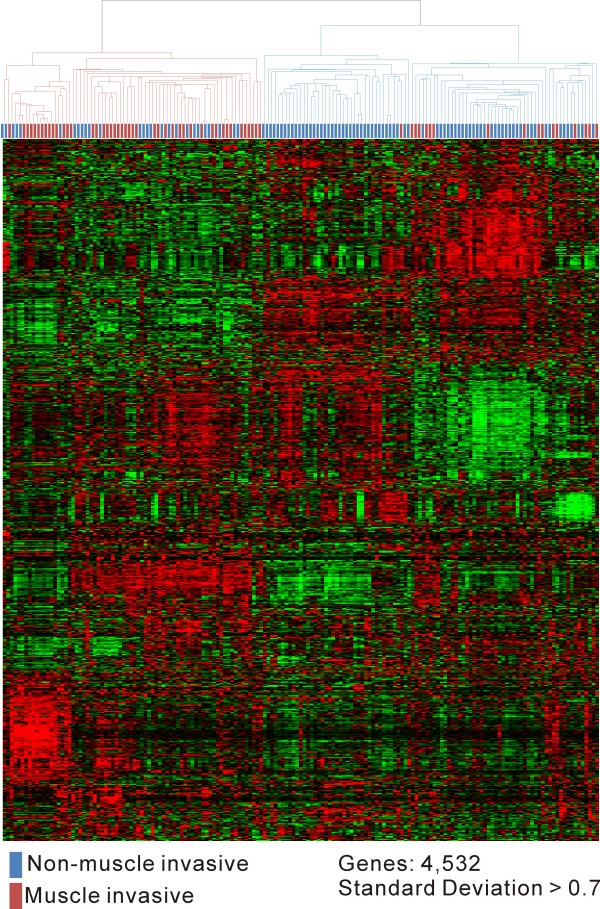
**Hierarchical cluster analysis of 165 primary bladder cancers (103 NMIBC and 62 MIBC)**. Genes with expression values that had a standard deviation of at least .7 were selected (4,532 genes). The red and green colors reflect high and low expression levels, respectively.

We performed microarray analysis of tumor tissue from 165 primary bladder cancer patients followed by unsupervised hierarchical clustering analysis. Tumors with similar repertoires of gene expression were grouped into two main clusters without knowledge of tumor class. The two main clusters exhibited a strong association with stage (Fig. 2).

### Selection of Prognosis-related Gene Classifiers Using Microarray Gene Expression Profiling

To select prognosis-related genes, a separate analysis was performed based on invasiveness and prognosis. In univariate Cox regression analysis, 42 genes were correlated significantly with recurrence and 97 with progression in NMIBC. Similarly, 44 genes were closely related with progression, 49 with cancer-specific survival, and 61 signatures with overall survival in MIBC (available at http://www.ncbi.nlm.nih.gov/geo/query/acc.cgi?acc=GSE13507). To evaluate the prognostic properties of each gene classifier, genes in the top 10th percentile for each classifier were chosen (total of 30 genes; 4 in recurrence-related gene classifier and 10 in progression-related gene classifier for NMIBC; 5 in progression-related gene classifier, 5 in cancer-specific survival-related gene classifier and 6 in overall survival-related gene classifier for MIBC). Risk scores were calculated for these selected genes, and the 50th percentile (median) value was used as the cutoff for each classifier to discriminate between good- or poor-prognosis signature groups [9,21-24].

**Figure 3:**
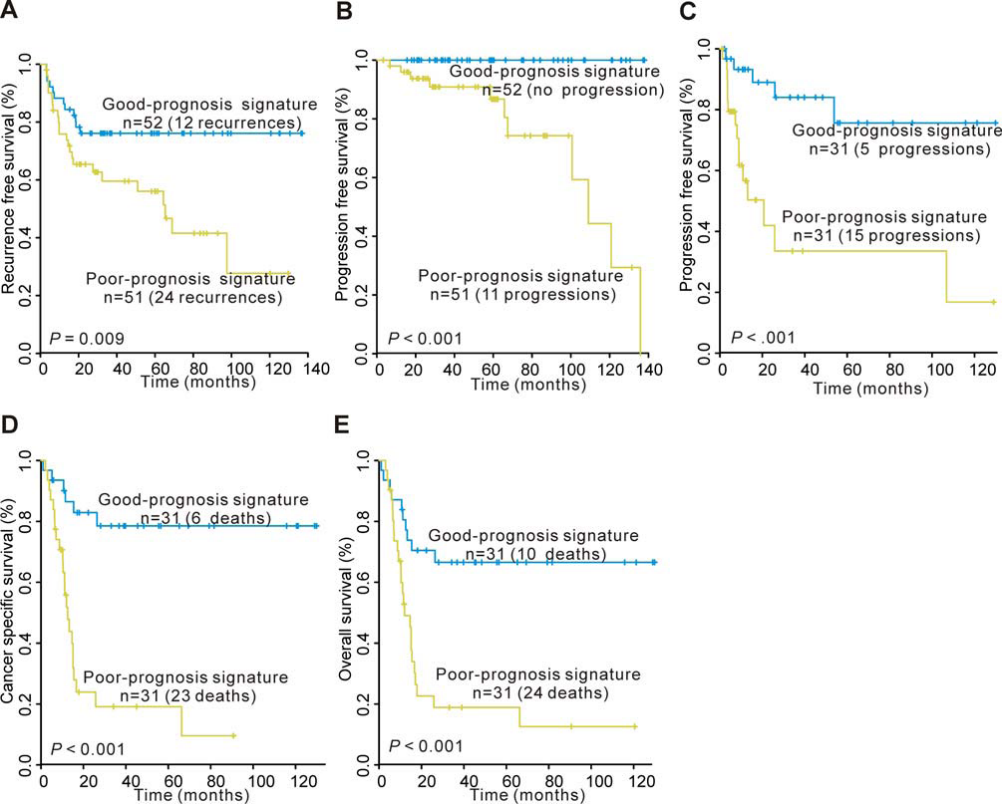
**Kaplan-Meier estimations in primary bladder cancer with gene signatures based on microarray analysis of the original training cohort**. Kaplan-Meier curves of (A) recurrence (B) and progression in NMIBC. Kaplan-Meier curves of (C) progression, (D) cancer-specific survival and (E) overall survival in MIBC.

Patients with NMIBC in the poor-prognosis signature group had a significantly shorter recurrence time than those in the good-prognosis signature group (P = 0.009; Fig. 3A). When we examined progression in NMIBC, the time to progression was shorter for patients with poor-prognosis signature than good-prognosis signature. Furthermore, none of the patients with good-prognosis signature were associated with progression (P < 0.001; Fig. 3B). Tumor progression, cancer-specific survival and overall survival in MIBC were significantly different between the two groups having good-, and poor-prognosis signatures, respectively (each P < 0.001; Fig. 3C-E).

### Validation of Gene Classifiers in the Original Cohort by RT-PCR

Selected gene classifiers were validated by RT-PCR. Of the 30 genes analyzed, 14 genes met our selection criteria for validation in the original cohort: 3 in recurrence-related gene classifier and 8 in progression-related gene classifier for NMIBC, and 3 in progression-related gene classifier for MIBC (Table 2). None of the cancer-specific and overall survival-related gene classifiers in MIBC exhibited a correlation coefficient of greater than .6 between the microarray and RT-PCR results. Thus, we were unable to validate these genes in the original cohort.

**Table 2:** Correlations Between Microarray Data and RT-PCR Data for 14 Selected Genes

Prognosis related-gene Classifiers	Gene	UniGene Number	Up/down*	Correlation^+^	*P*^‡^
Recurrence related-gene classifier in NMIBC	MGC34830	Hs.502266	Up	0.72	< 0.001
	FANCB	Hs.554740	Up	0.62	< 0.001
	CASP8AP2	Hs.558218	Up	0.66	< 0.001
Progression related-gene classifier in NMIBC	S100A8	Hs.416073	Up	0.85	< 0.001
	CELSR3	Hs.631926	Up	0.85	< 0.001
	PFKFB4	Hs.476217	Up	0.72	< 0.001
	HMOX1	Hs.517581	Up	0.69	< 0.001
	MTAP	Hs.193268	Down	0.81	< 0.001
	MGC17624	Hs.461655	Down	0.68	< 0.001
	KIF1A	Hs.516802	Up	0.72	< 0.001
	COCH	Hs.21016	Up	0.78	< 0.001
Progression related-gene classifier in MIBC	CDH3	Hs.709226	Up	0.74	< 0.001
	DSC3	Hs.41690	Up	0.67	< 0.001
	PPP1R14C	Hs.486798	Up	0.62	< 0.001

Abbreviations: NMIBC, non-muscle invasive bladder cancer; MIBC muscle invasive bladder cancer.

* Up-regulated or down-regulated in poor prognosis group.

^+ ^Correlation coefficient for microarray values versus RT-PCR values.

‡ *P *values for the correlation coefficients were calculated by Pearson correlation coefficient test.

**Figure 4:**
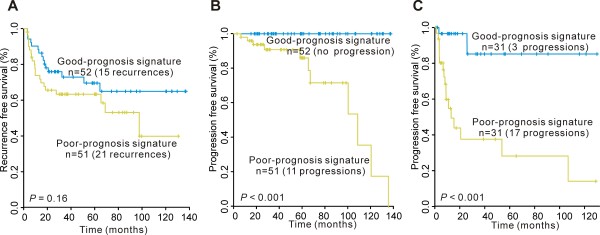
**Kaplan-Meier estimations in primary bladder cancer with gene signatures based on RT-PCR analysis of the original validation cohort**. Kaplan-Meier curves of (A) recurrence and (B) progression in NMIBC, and (C) progression in MIBC.

Patients with good-prognosis signature exhibited an increased time to recurrence than patients with poor-prognosis signature in NMIBC, but this difference was not statistically significant (P = 0.16; Fig. 4A). Analysis of progression-related gene classifier in NMIBC revealed significant differences in time to progression between the good-prognosis and poor-prognosis signature groups (P < 0.001; Fig. 4B). Notably, for NMIBC, no patient with good-prognosis signature was associated with disease progression. Thus, multivariate analysis of disease progression could not be carried out due to the fact that none of the patients in the good-prognosis signature group were associated with progression. Consistent with the microarray data for progression-related gene classifier in MIBC, the good-prognosis signature group had a prolonged time to progression as compared to the poor-prognosis signature group (P < 0.001; Fig 4C).

### Independent Validation by RT-PCR

**Figure 5:**
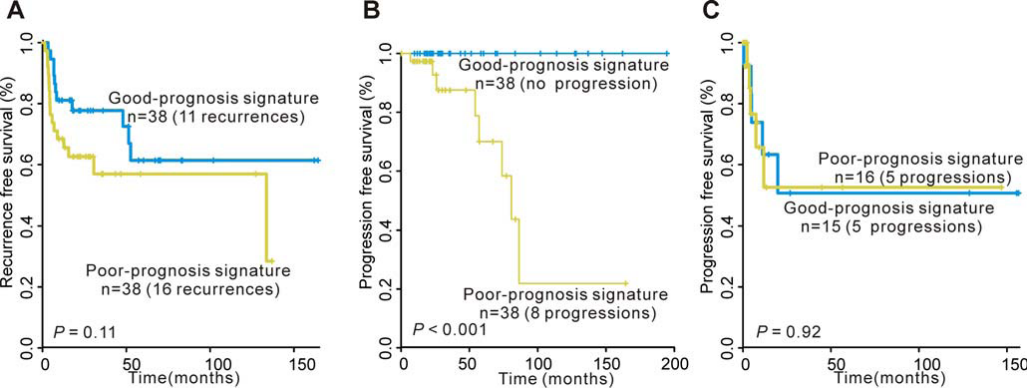
**Kaplan-Meier estimations in primary bladder cancer with gene signatures based on RT-PCR analysis of the independent validation cohort**. Kaplan-Meier curves of (A) recurrence and (B) progression in NMIBC, and (C) progression in MIBC.

We used RT-PCR to validate prognosis-related gene classifiers (3 in recurrence-related gene classifier and 8 in progression-related gene classifier for NMIBC and 3 in progression-related gene classifier for MIBC) in an independent cohort of 107 primary bladder cancer patients. As with the original cohort, risk scores were calculated and a median value was used to differentiate the risk groups. For NMIBC, the good-prognosis signature group had a significantly increased time to progression as compared to the poor-prognosis signature group (P < 0.001; Fig. 5B). As with the original cohort, none of the patients with good-prognosis signature were associated with progression for NMIBC. Recurrence-related gene classifier in NMIBC and progression-related gene classifier in MIBC did not uncover any differences in respective time to recurrence and progression between good- and poor-prognosis signature groups in the independent cohort (each P >0.05; Fig. 5A, C).

## Discussion

Although there have been reports of the value of gene expression profiles for cancer prognosis [8-12], only limited data are available on the prognostic value of expression profiles in human bladder cancer in relatively large-scale study populations with long-term follow-up [25-27]. With regard to the microarray studies that have been performed in the bladder cancer field to date, Sanchez-Carbayo et al. [25] used cDNA microarrays to facilitate the classification scheme of diagnostic and prognostic utility for stratifying advanced bladder cancer. This technology allowed them to identify poor outcome profile could assist in selecting patients who may benefit from more aggressive therapeutic intervention. Additionally, Dyrskjot et al. [26,27] also reported the clinical usefulness of molecular markers for the prediction the clinical course of patients with NMIBC. These studies enhance the importance of the genome-wide studies in bladder cancer fields. In concordance with earlier reports, our study should support the potential usefulness of microarray study in these fields [25-27]. However, both the validity and the reproducibility of microarray-based clinical research have been challenged [28]. To develop and validate a method of classification, it is necessary to start with a sufficiently large set of samples to analyze an independent test set and a validation set [5]. Furthermore, the results of microarray analysis in general should be validated using other techniques for quantifying RNA expression and by an independent cohort to reduce the false discovery rate [29,30]. In the present study, we used microarray data to identify several types of prognosis-related gene classifiers. We were able to validate progression-related gene classifier in primary NMIBC by RT-PCR in a relatively large-scale long-term follow-up independent study population, but not for the other prognosis-related gene classifiers. These findings emphasize the importance of validation to support the reliability of microarray-based results.

The prediction of disease progression for patients with bladder cancer is a major clinical challenge. A number of molecular markers of cancer that have been identified to date have been explored as predictors of disease progression. Some of these, such as p53, have been suggested to be independent markers, while others do not appear to have a role as prognostic indicators [1-3]. Because multiple genetic alterations are required for the transformation of a normal cell into a cell with a malignant and ultimately metastatic phenotype, assessment of multiple markers as a whole might better describe the biological phenotype of a particular cancer.

We identified the following eight genes as predictors of progression in patients with NMIBC: S100 calcium binding protein A8 (S100A8), 6-phosphofructo-2-kinase/fructose-2,6-biphosphatase 4 (PFKFB4), heme oxygenase 1 (HMOX1), methylthioadenosine phosphorylase (MTAP), kinesin family member 1A (KIF1A), coagulation factor C homolog (COCH), EGF LAG seven-pass G-type receptor 3 (CELSR3) and chromosome 16 open reading frame 74 (MGC17624). Overexpression of S100A8 is associated with stage progression, invasion, metastasis and poor survival in human bladder cancer [31]. Thus, the expression of S100A8 in NMIBC could be an early indicator for a sub-group of tumors with a propensity for muscle invasion. PFKFB4 is strongly induced by hypoxia through an hypoxia inducible factor 1 alpha (HIF1A) subunit dependent mechanism, and might contribute significantly to the Warburg effect observed in malignant gastric, pancreatic, breast and colon tumors [32,33]. HMOX1 is an essential enzyme in heme catabolism. HMOX1 participates in the processes of angiogenesis and vasculogenesis [34]. There are significant differences in the distribution of the HMOX1 genotype in patients with different-stage urothelial carcinoma [35]. MTAP encodes an enzyme that plays a major role in polyamine metabolism. Many cancers, including bladder cancer, are deficient of the MTAP enzyme because the gene is co-deleted along with the tumor suppressor p16 [36]. KIF1A, a member of the kinesin family, appears to play a critical role in the development of axonal neuropathies resulting from impaired axonal transport [37]. However, the function of KIF1A related to cancer is unknown. COCH is a cell adhesion molecule [38]. The specific functions of CELSR3 and MGC17624 have not been determined. Although the identification of genes linked to disease progression suggests potential therapeutic interventions based on their mechanism of action, the lack of a biological context for these genes does not diminish their potential as clinical biomarkers. Many biomarkers, such as prostate-specific antigen and carcinoembryonic antigen, have unknown functions but are nonetheless useful as diagnostic or prognostic markers for disease [5].

Because some genetic events occur early in the disease process or before progression, molecular diagnosis may enable the prediction of disease progression before the onset of symptoms or overt radiographic evidence. Thus, from a clinical point of view, the most promising application for gene classifiers is the early prediction of tumor progression. In addition, new relevant biomarkers must provide cost-effective, non-invasive monitoring of low-risk patients and identify high-risk refractory tumors before they progress. The main goal of all of these efforts is to develop more accurate prognostic tools that can be used to direct treatment [5]. In the current study, we identified a specific subset of genes that predicted progression in NMIBC. NMIBC is a heterogeneous group including patients with the enormous array of clinical and pathological risk factors involved, such as number of tumors, tumor size, prior recurrence rate, T-category, carcinoma in situ, grade, intravesical therapy, and other factors [2,3,39]. Thus, multivariate analysis including these factors needs to control confounding effects. However, in the present study, multivariate analysis of disease progression could not be carried out due to the fact that any patients with NMIBC in the good-prognosis signature group did not experience cancer progression. Nonetheless, this gene set of progression-related markers has favorable potential to contribute to the understanding of bladder cancer behavior and the future treatment of patients with urothelial cancer.

## Conclusions

The progression-related gene classifier identified in the current study represents a potentially valuable tool for the stratification of heterogeneous bladder cancer patient populations into risk groups that can be used to guide clinical decision-making, including observation versus adjuvant therapy. We derived clear clinical results using gene classifier for progression in NMIBC. The results of the current study suggest that the gene expression profiling to determine progression can be used to provide customized clinical care.

## Competing interests

The authors declare that they have no competing interests.

## Authors' contributions

WJK designed, carried out experiments, interpreted and analyzed the results and wrote the manuscript, EJK, YSH, PJ, MJK, SJY, SCL participated in the experiments, SKK, YJK designed, interpreted and analyzed the results and wrote the manuscript, KML, SKM, EJC and SCB assisted with the draft of the manuscript and critically revised the manuscript. All authors read and approved the final manuscript.
